# Role of Adenosine Deaminase Estimation in Differentiation of Tuberculous and Non-tuberculous Exudative Pleural Effusions

**DOI:** 10.4021/jocmr2010.03.280w

**Published:** 2010-03-31

**Authors:** Bharat Kumar Gupta, Vinay Bharat, Debapriya Bandyopadhyay

**Affiliations:** a Department of Biochemistry, Subharti Medical College, S. V. S. University, Meerut, India; b Department of Pathology, Subharti Medical College, S. V. S. University, Meerut, India

## Abstract

**Background:**

Tuberculosis kills five lakh patients every year in India, commonest being pulmonary tuberculosis and is often associated with effusion. Delay in diagnosis and treatment results in poor prognosis. Several studies have suggested the role of adenosine deaminase (ADA) in the diagnosis of tuberculous pleural effusions, but false-positive results from lymphocytic effusions have also been reported. The purpose of this study is to find out the role of ADA levels in differentiation of tuberculous and non-tuberculous exudative pleural effusions of different etiologies.

**Methods:**

Ninety-six lymphocytic pleural fluid samples were consecutively selected and divided into two groups: tuberculous (n = 56) and non-tuberculous (n = 40), depending upon the etiology [Malignancy (n = 16), Infectious diseases (n = 18), Pulmonary embolism (n = 1), Collagen vascular diseases (n = 3) and Sarcoidosis (n = 2)]. ADA was estimated in pleural fluid in all the cases.

**Results:**

In all 56 samples, ADA level of tuberculous group was above diagnostic cut-off (40 U/L), while only one sample was above cut-off in non-tuberculous group (2.5%). The negative predictive value of ADA for the diagnosis of non-tuberculous etiology was 97.5% (39 of 40) lymphocytic pleural effusion patients.

**Conclusions:**

In this study, ADA levels in nontuberculous exudative pleural effusions rarely exceeded the cut-off; set for tuberculous disease. The pleural fluid ADA levels were significantly higher in tuberculous exudative pleural effusions when compared with non-tuberculous exudative pleural effusions.

**Keywords:**

Adenosine deaminase; Tuberculous effusion; Pleural fluid; Exudative pleural effusions

## Introduction

Five lakh patients of tuberculosis die every year in India [1], commonest being pulmonary tuberculosis and is often associated with effusion, of which 8.3 % is childhood tuberculosis. Multidrug resistance in tuberculosis and its association with acquired immuno-deficiency syndrome (AIDS) has further highlighted the importance of this disease. Delay in diagnosis and in the start of effective treatment results in poor prognosis and sequalae in upto 25% of cases [2]. Available methods of diagnosis of tuberculosis were evaluated and all of them were found to have low sensitivity and specificity. Direct evidence of acid fast bacilli (AFB) is available only in small percentage of cases.

Similar difficulties are encountered in cases of tuberculous pleuritis (TP). TP with effusion as a complication of primary pulmonary tuberculosis has been reported to occur in 2 to 38% of children with pulmonary disease, but it is more likely to occur in adolescents and adults [3-9]. The pleural fluid of TP is usually predominantly lymphocytic. Some studies suggest that pleural fluid lymphocyte percentages of more than 85% are very suggestive of tuberculosis [10], while the other series of patients with TP indicates that only 10% patients had less than 50% lymphocytes in pleural fluid [11]. Acute TP may show an increase in neutrophils [12]. But if a continuous pleural tapping is done, the cell count shows change. Apart from tuberculosis the other causes of lymphocytic pleural effusions include malignancies, infectious diseases, pulmonary embolism, collagen vascular diseases, sarcoidosis, uremia, chylothorax and post-coronary artery bypass graft (CABG) pleural effusion [13].

The classification of pleural effusion into exudative and transudative to support the clinical diagnosis has been well established. There are several causes of exudative pleural effusion including tuberculosis, neoplasms (primary or secondary), pyogenic bacterial infections, fungal infection, sarcoidosis, collagen vascular disease, transplant patients with graft rejection, trauma, and pulmonary embolism. Microscopy of fluid shows neutrophillic predominance in bacterial infectious etiology, while in others there is lymphocytic predominance. Presentation of pulmonary embolism can be obscure either as a transudative or an exudative pleural collection.

Usefulness of adenosine deaminase (ADA) estimation in pleural fluid has been shown as a reliable chemical biomarker specially when there is suspicion of tuberculosis in endemic areas. Sometimes the increase is marked in early stages of the disease and in some other conditions with neutrophillic effusions like in parapneumonic and empyema [14]. Researchers have established that ADA level rarely exceeds the cut-off set for tuberculous effusion in non-tuberculous lymphocytic effusions [15].

The purpose of this study is to find out the role of ADA estimation in differentiation of tuberculous and non-tuberculous exudative pleural effusions.

## Materials and Methods

The project was approved by institutional ethical committee. Patients with pleural effusion admitted in the wards of Tuberculosis and chest diseases department from January 08 to November 09 at the Subharti Medical College, Meerut, India were included in the present study. After the routine procedure of informed consent, history taking and examination, routine investigations were done in all the patients.

Presence of first or more than one of the following criteria was adopted to label a case as tuberculous: (1) bacteriological confirmation of presence of Mycobacterium tuberculosis (direct smear or culture or histological finding); (2) histopathology finding of caseating granulomas; (3) radiological findings consistent with TB; (4) clinical presentation consistent with TB with exclusion of other clinical considerations; (5) definite clinical and radiological improvement in two months of administration of exclusive anti-tubercular treatment; (6) history of contact of current disease and positive reaction (> 20 mm induration) to the 5 tuberculin unit (TU) purified protein derivative (PPD).

Pleural tap was done in all the cases and fluid tested for pH, glucose, proteins, LDH, total ADA, microscopy, cytology and microbial testing (Grams staining, Z N Staining, cultures). Pleural biopsy was done in selected cases of tuberculosis and malignancy.

All the cases had exudative effusion as per Lights criteria (pleural fluid protein / serum protein > 0.5; fluid LDH / serum LDH > 0.6) [16].

Effusion was called as malignant when pleural fluid cytology or pleural biopsy showed evidence of malignancy or if the patient had proved metastatic malignancy with no other detectable cause of effusion. In infectious disease effusion, patient had a history of fever and pulmonary infiltrate with complete response to antibiotic therapy; pulmonary embolism effusion patients presented with typical dyspnoea of sudden onset, leg pains with its signs and symptoms; collagen vascular disease effusion patients had a history of fever and dyspnoea with evidence of eosinophilia and arrhythmia while sarcoidosis effusion patients had multi organ involvement with presence of non-caseating granuloma.

Adenosine deaminase estimation was done by Blake-Berman method [17] which is comparable with the Giusti-Galanti method as is established by a meta-analysis of 2251 cases [18]. Adenosine deaminase Level of more than 40 U/L is taken as cut-off for tuberculosis, same value was taken by other workers also [19].

### Statistical analysis

Results are expressed as Mean ± SD. The statistical analyses applied included the standard normal variate test, to analyze the difference between tubercular (n = 56) and non-tubercular (n = 40) quantitative variables at 1% level of significance (Table 4).

## Results

In our study, 96 samples were thus included between the age group of 12 to 76 years. Male : Female ratio is 3 : 1. Tuberculous group had 56 samples while non-tuberculous group had 40 samples. Non-tuberculous group included different etiologies, namely, malignancy (n = 16), infectious diseases (n = 18), pulmonary embolism (n = 1), collagen vascular diseases (CVD) (n = 3) and sarcoidosis (n = 2) (Table 1).

**Table 1:** Distribution of the Cases According to Accepted Criteria

Group	Disease	Number of Cases
Tuberculosis (n = 56)	Tuberculosis	56
Non-Tuberculosis (n = 40)	Malignancy	16
	Infectious diseases	18
	Pulmonary embolism	1
	Collagen vascular disease	3
	Sarcoidosis	2

Out of 56, only 9 cases (16.07%) were confirmed as having tuberculosis by bacteriological confirmation of presence of Mycobacterium tuberculosis.

Mean ± SD of pH in tuberculous group was 7.31 ± 0.09, in malignant group it was 7.32 ± 0.07, in infectious disease group it was 7.31 ± 0.08, in pulmonary embolism it was 7.29, in CVD group it was 7.27 ± 0.05 while in sarcoidosis it was 7.32 ± 0.03 (Table 2).

Mean ± SD of glucose in tuberculous group was 46.2 ± 17.6, in malignant group it was 42.2 ± 19.6, in infectious disease group it was 31.4 ± 17.8, in pulmonary embolism it was 40.7, in CVD group it was 51.5 ± 14.6 while in sarcoidosis it was 58.8 ± 18.9 mg % (Table 2).

Mean ± SD of protein in tuberculous group was 4.3 ± 0.9, in malignant group it was 4.2 ± 1.1, in infectious disease group it was 4.1 ± 1.2, in pulmonary embolism it was 3.9, in CVD group it was 4.4 ± 0.7 while in sarcoidosis it was 4.2 ± 0.9 gm % (Table 2).

Mean ± SD of LDH in tuberculous group was 151.6 ± 27.0, in malignant group it was 147.4 ± 18.6, in infectious disease group it was 141.7 ± 23.1, in pulmonary embolism it was 136.0, in CVD group it was 154.6 ± 13.7 while in sarcoidosis it was 148.6 ± 11.6 U/L (Table 2).

**Table 2:** Biochemical Parameters in Pleural Fluid in Different Groups

Group	Disease	pH	Glucose mg%	Protein g%	LDH U/L
Tubercular (n = 56)	Tuberculosis (n = 56)	7.31 ± 0.09	46.2 ± 17.6	4.3 ± 0.9	151.6 ± 27.0
Non-Tubercular (n = 40)	Malignant (n = 16)	7.32 ± 0.07	42.2 ± 19.6	4.2 ± 1.1	147.4 ± 18.6
	Infectious diseases (n = 18)	7.31 ± 0.08	31.4 ± 17.8	4.1 ± 1.2	141.7 ± 23.1
	Pulmonary embolism (n = 1)	7.29	40.7	3.9	136.0
	Collage Vascular Disease (n = 3)	7.27 ± 0.05	51.5 ± 14.6	4.4 ± 0.7	154.6 ± 13.7
	Sarcoidosis (n = 2)	7.32 ± 0.03	58.8 ± 18.9	4.2 ± 0.9	148.6 ± 11.6

In tuberculous group the range of ADA was 115.4 (40.6 - 156.0) and mean ± SD was 67.34 ± 22.85 while collectively in non-tuberculous group it was 33.2 (4.8 - 38.0) and 18.60± 9.12 (Table 3).

Amongst different groups in non-tuberculous disease: in malignant group range of ADA was 26.6 (6.4 - 33.0) and mean ± SD was 9.08 ± 9.96, in infectious disease group its range was 27.2 (4.8 - 32.0) and mean ± SD was 18 ± 7.83, in pulmonary embolism it was 13.58, in CVD group its range was 5 (10.0 - 15.0) and mean ± SD was 12 ± 2.65, in sarcoidosis group its range was 8 (30.0 - 38.0) and mean ± SD was 34 ± 5.66 U/L (Table 3).

**Table 3:** ADA Range With Mean ± SD in Different Groups

Group	Disease	Range	Mean ± SD
Tubercular (n = 56)	TB (n = 56)	115.4 (40.6 - 156.0)	67.34 ± 22.85
Non-Tubercular (n = 40)	Over all	33.2 (4.8 - 38.0)	18.60 ± 9.12
	Malignant (n = 16)	26.6 (6.4 - 33.0)	9.08 ± 9.96
	Infectious diseases (n = 18)	27.2 (4.8 - 32.0)	18 ± 7.83
	Pulmonary embolism (n = 1)	13.58	--
	Collagen Vascular Disease (n = 3)	5 (10.0 - 5.0)	12 ± 2.65
	Sarcoidosis (n = 2)	8 (30.0 - 38.0)	34 ± 5.66

Adenosine deaminase level in all 56 samples of tuberculous group was above diagnostic cut-off (40 U/L); while in non-tuberculous group only one sample was above cut-off (2.5%). The negative predictive value of ADA for the diagnosis of non-tuberculous etiology was 97.5% (39 of 40) exudative pleural effusion patients (Table 4).

Adenosine deaminase values were compared between tuberculous and non-tuberculous groups and difference in these values was statistically highly significant. On comparing the ADA values amongst different non-tubercular groups; the difference was not statistically significant (Table 4).

**Table 4:** Pleural Fluid ADA Levels With Their Significance

Group	Number of Cases	ADA Levels in U/L	True Positive / Negative Cases	Mean ± SD	z cal	P-value
Tuberculous	56	> 40	56	67.34 ± 22.85	14.48	0.0007 (p < 0.01)*
		≤ 40	00			
Non-tuberculous	40	> 40	01	18.60 ± 9.12		
		≤ 40	39			
Total	96		96			

*p < 0.01 shows a highly significant difference between the tuberculous and non-tuberculous groups.

## Discussion

Tuberculous pleurisy (TP) is a result of delayed hypersensitivity reaction to the tubercle bacilli. Exudative pleural effusion is manifested by various diseases. The diagnosis based on pleural tap: AFB staining is positive in only 10 to 25% of the cases, while culture for AFB is positive in less than 25% of the cases [20]. Perez et al in their study have shown the positivity for tubercle bacilli with smear alone in pleural fluid is 11.1% [21], with culture it is 33.3% and with pleural biopsy it increases up to 96.2%. These values differ among different workers [22, 23]. One third of patients with this condition can have a negative tuberculin skin test [11]. Sensitive techniques like PCR show positive results in about 50% of cases [24].

In patients with tuberculous exudative pleural effusion, neutrophils predominate in the early stages of the disease, while abundant mononuclear cells is a classical finding later and is believed to be due to the proliferation and differentiation of lymphocytes which release lymphokines, which in turn activate macrophages for an enhanced bactericidal activity [25, 26]. Even pleural fluid cytology takes a back seat while investigating the cause of an exudative pleural effusion, and is usually just evidence supporting our final diagnosis.

Since the conventional diagnostic tools are incapable of pinpointing the cause, so several bio-markers like ADA, interferon (IFN)-γ, a variety of tumor markers and cytokines, and C-reactive protein (CRP) have been proposed as alternative noninvasive means of establishing tuberculous etiology in cases of exudative pleural effusion [27].

Adenosine deaminase estimation in pleural fluid has long been taken as a marker for tuberculous pleurisy. Levels above 40 U/L indicate pleural tuberculosis with sensitivity 81 to 100% and specificity 83 to 100% [28-30], while some other workers have observed that this cut-off indicates a still higher sensitivity of 90 - 100% and specificity of 89 - 100% [29, 31-35]. False positive cases reported could be due to empyema, lymphoma, malignancy, parapneumonic or collagen vascular disease [29, 36].

The ADA is an enzyme involved in the purine catabolism. It catalyzes the deamination of adenosine to inosine and of deoxyadenosine to deoxyinosine. Adenosine deaminase is involved in the proliferation and differentiation of lymphocytes, specifically the T-lymphocytes. The T-cells release ADA during the process of activation in the presence of live intracellular pathogens. Thus ADA has been looked upon as a marker of cell mediated immune response and specifically T-cell activation. There are several known isoforms of ADA, which arise from different gene loci [37] out of which ADA-1 is found in all cells including lymphocytes and monocytes, while ADA-2 is found exclusively in monocytes [38]. ADA-2 isoform is the one raised in tuberculous pleurisy, accounting for almost 88% of total ADA activity. Rise in ADA-1 activity is more commonly associated with pyogenic bacterial infection of the pleural cavity, contributing to a median 70% of total ADA activity [34]. However there is no clear advantage of using the ADA-2 over the total ADA activity in clinical practice [25]. The total ADA activity assay is in fact preferred for its rapid turnover and low cost.

Daniil et al, in their study, concluded that the combination of pleural ADA and CRP levels might be sufficient for discriminating between the different groups of exudative pleural effusion [27]. Jadhav and Bardapurkar concluded that ADA is a useful biochemical marker to evaluate exudative pleural effusions [39].

We have found the pleural fluid ADA levels to be consistently increased and more than the cut-off (40U/L) in cases of exudative pleural effusions of tuberculous etiology. In cases with non-tubercular exudative pleural effusion the ADA levels were found to be consistently below the cut-off. The negative predictive value of ADA for the diagnosis of non-tuberculous etiology was 97.5%. Therefore, pleural fluid ADA levels can play a very significant role in differentiating cases of exudative pleural effusion into tuberculous and non-tuberculous.

On the contrary, Rafael concluded that the ADA assay should be considered as a screening test to guide further diagnostic procedures in cases of exudative pleural effusion [40]. While Kataria and Imtiaz concluded that ADA testing can be an integral part of diagnostic workup in lymphocyte rich exudative body fluids both in countries with high and low prevalence of tuberculosis [25].

The sensitivity of the ADA as a serological marker for tuberculous exudative pleural effusion depends on the prevalence of the disease in the population. In western countries where the prevalence of tuberculosis is low, the positive predictive value of the test decreases because of the high false positives, but the negative predictive value remains high. Thus ADA can be used for ruling out suspected cases of tuberculosis and can be a very effective screening test.

India has a high prevalence of tuberculosis and the sensitivity and specificity of this test will be high in this population. Therefore ADA estimation being a simple, low cost, rapid and non-invasive test, should become an integral part of the diagnostic work up of exudative pleural effusions in suspected cases of tuberculosis.

In conclusion, in this study, ADA levels in non-tuberculous exudative pleural effusions rarely exceeded the cut-off; set for tuberculous disease. The pleural fluid ADA levels were significantly higher in tuberculous exudative pleural effusions when compared with non-tuberculous exudative pleural effusions. Adenosine deaminase level of less than 40 U/L practically excludes the tubercular etiology in exudative pleural effusion cases and thus may be useful in differentiating tuberculous etiology from others in exudative pleural effusion.
